# Migration patterns and hybridization within the Asian stonechat complex in response to a major geographical barrier

**DOI:** 10.1093/evlett/qrag019

**Published:** 2026-05-05

**Authors:** Tianhao Zhao, Yury Anisimov, Wieland Heim, Gang Song, Valentina Anisimova, Nyambayar Batbayar, Christen M Bossu, Andrea Bours, Siqiao Chen, Wenjia Chen, Batmunkh Davaasuren, Dule, Shengjing Jiao, Xiaolu Jiao, Magnus Hellström, Georg Langebrake, Zhuo Li, Shu-Yueh Liao, Aitao Liu, Zongzhuang Liu, Jacob Roved, Xinyuan Wang, Matthias H Weissensteiner, Guannan Wen, Dezhi Zhang, Guoming Zhang, Yifang Zhang, Kristen Ruegg, Miriam Liedvogel, Staffan Bensch, Bregje Wertheim, Fumin Lei, Barbara Helm

**Affiliations:** Groningen Institute for Evolutionary Life Sciences (GELIFES), University of Groningen, Groningen, The Netherlands; Bukhara, Uzbekistan; Institute for Biology and Environmental Sciences, University of Oldenburg, Oldenburg, Germany; Institute of Zoology, Chinese Academy of Science, Beijing, China; Department of Natural Sciences of the Pedagogical Institute, Irkutsk State University, Irkutsk, Russia; Wildlife Science and Conservation Center of Mongolia, Ulaanbaatar, Mongolia; Department of Biology, Colorado State University, Fort Collins, United States; Max Planck Institute for Evolutionary Biology, Plön, Germany; Kunming, Yunnan, China; Department of Ecology and Evolution, University of Copenhagen, Copenhagen, Denmark; Wildlife Science and Conservation Center of Mongolia, Ulaanbaatar, Mongolia; Hohhot, Inner Mongolia, China; Department of Agriculture and Biology, Shanghai Jiao Tong University, Shanghai, China; Institute of Zoology, Chinese Academy of Science, Beijing, China; Ottenby Bird Observatory, BirdLife Sweden, Ottenby, Sweden; Institut für Vogelforschung “Vogelwarte Helgoland”, Wilhelmshaven, Germany; School of Ecology and Environmental Science, Yunnan University, Kunming, China; Department of Natural Resources and Environmental Sciences, University of Illinois at Urbana-Champaign, Urbana, United States; Department of Biology, Colorado State University, Fort Collins, United States; Department of Ecology and Genetics, Uppsala University, Uppsala, Sweden; Global Institute, Copenhagen University, Copenhagen, Denmark; Changchun, Jilin, China; Institut für Vogelforschung “Vogelwarte Helgoland”, Wilhelmshaven, Germany; Max Planck Institute for Evolutionary Biology, Plön, Germany; Institute of Zoology, Chinese Academy of Science, Beijing, China; College of Eco-Environmental Engineering, Qinghai University, Xining, China; School of Biological Resources and Environmental Sciences, Jishou University, Zhangjiajie, China; Department of Biology, Colorado State University, Fort Collins, United States; Institut für Vogelforschung “Vogelwarte Helgoland”, Wilhelmshaven, Germany; Department of Biology, Lund University, Lund, Sweden; Groningen Institute for Evolutionary Life Sciences (GELIFES), University of Groningen, Groningen, The Netherlands; Laboratory of Entomology, Wageningen University, Wageningen, The Netherlands; Institute of Zoology, Chinese Academy of Science, Beijing, China; Department of Bird Migration, Swiss Ornithological Institute, Sempach, Switzerland

**Keywords:** individual tracking, geographical barrier, intermediate route, speciation, hybrid zone, migratory divide

## Abstract

Facing geographical barriers, phenotypic responses of migratory landbirds may differ even between closely related taxa: some may bypass barriers while others cross them. Such phenotypic variation can arise from different genetic backgrounds and lead to different adaptive processes. A classic example is migratory divide, i.e., breeding ranges where different migration-route phenotypes co-exist or meet. Such variation can facilitate an understanding of the evolutionary background of migration and responses to barriers. In Asia, a migratory divide has been suggested in response to the major landscape, the Qinghai-Tibet Plateau (QTP) for 2 stonechat taxa (Siberian stonechat *Saxicola maurus maurus* and Amur stonechat *S. stejnegeri*). Detouring along either side of the QTP, these taxa are believed to disfavor crossing over the highland. However, the Tibetan stonechat (*S. m. przewalskii*), breeding on the QTP, may differ in its barrier response due to possible adaptation to high elevation. To investigate migration patterns and potentially associated genetic differences, we studied migration routes and population genetics of populations around the assumed migratory divide in Russia and Mongolia and of a Chinese population from the QTP. Our results confirmed the existence of a migratory divide between *maurus* and *stejnegeri*, albeit with extensive hybridization over generations. Of the 3 genotyped individuals employing intermediate routes across the QTP, 2 were clear hybrids. Meanwhile, *przewalskii* consistently followed a highland-crossing route and was genetically differentiated from *maurus* and *stejnegeri*. The diverse migration routes indicate that Asian stonechats respond differently to the geographical barrier. The partly viable intermediate route may be associated with hybridization and potentially facilitating gene flow between *maurus* and *stejnegeri*. The Asian stonechat complex thus offers great opportunities for research, with its specific evolutionary background associated with inhabiting and crossing the QTP, offering novel perspectives on the genetics and evolution of migration.

## Teaser

Migratory divides can be found in birds whose migration routes run across geographical barriers. Hybrids of parental types that employ alternative routes are hypothesized to embark on intermediate routes that would expose them to suboptimal conditions, resulting in post-zygotic reproductive isolation. However, to what extent geographical features function as barriers depends on a species’ physical abilities. In species whose sister taxon actually breeds on the barrier, its prohibitive effects may be reduced. This is the case in the Asian stonechat complex breeding near or on the Qinghai-Tibet Plateau (QTP). We demonstrated a migratory divide in central Siberia to Mongolia for the taxa *maurus* and *stejnegeri*, yet showed also evidence for extensive hybridization. Intermediate route was also documented, which seemed viable and overlapped with the route of *przewalskii* over the QTP. This Asian migratory divide in stonechats thus provided new nuance on the evolution of landbird migration.

Migration behavior exhibits a wide diversity across avian taxa, encompassing its occurrence, timing, direction, routes, and distances. Our accumulated knowledge on migration patterns has revealed substantial inter- and intra-specific variation, which is often associated with genetic differences and evolutionary histories (Justen & Delmore, 2022; Liedvogel et al., 2011; Ruegg et al., 2017).

Migratory divides, i.e., areas where two closely related taxa that exhibit different migration routes co-exist, potentially meet, mate, and likely also hybridize, are focal points of spatial diversity during migration. Such migratory divides have been documented in birds of all major global flyways, e.g., in the European-African Flyway within willow warblers (*Phylloscopus trochilus*) and Eurasian blackcaps (*Sylvia atricapilla*) (Bensch et al., 1999, 2009; Caballero‐López et al., 2021; Delmore et al., 2020b; Helbig, 1996; Helbig et al., 1994; Sokolovskis et al., 2023); in the Americas Flyway within Swainson’s Thrush (*Catharus ustulatus*) (Ruegg & Smith, 2002); in the Atlantic Flyway within Red-necked Phalarope (*Phalaropus lobatus*) (van Bemmelen et al., 2019); and in the Central Asian Flyway within the Barn Swallow (*Hirundo rustica*) (Scordato et al., 2020; Turbek et al., 2022). There is also a presumed east-west migratory divide in central Siberia described in, for example, the Siberian/Amur Stonechats (*Saxicola maurus, S. stejnegeri*), Citrine Wagtail (*Motacilla citreola*), and Siberian Bluethroat (*Luscinia svecica*) (Irwin et al., 2005b; Strubbe et al., 2025).

It has been frequently proposed that phenotypic variation in migration behaviors, especially within migratory divides, is associated with geographical barriers, for example, the Sahara Desert, the Mediterranean Sea, the Rocky Mountains, and the Qinghai-Tibet Plateau (QTP) (Irwin et al., 2005b; Justen et al., 2024; Sokolovskis et al., 2023; Turbek et al., 2022; Zhao et al., 2020, 2024). Instead of facing the various challenges when flying directly across the barrier regions, e.g., from weather, ground elevation, lack of roosting, and refueling sites (Bishop et al., 2015; Irwin et al., 2005b; Sjöberg et al., 2021), birds commonly bypass these barriers along longer detours in different flyways (Ruegg & Smith, 2002; Sokolovskis et al., 2023; Turbek et al., 2022; Zhao et al., 2024). However, some avian taxa also migrate directly across barriers (Hawkes et al., 2011; Lindström et al., 2021; Sjöberg et al., 2021; Yu et al., 2024).

An alternative scenario for the formation of migratory divides was proposed by Irwin et al. (2005b), based on ample evidence for partial genetic control of migration routes (Justen & Delmore, 2022; Liedvogel et al., 2011). In their biogeographic model, the different migration routes were formed independently. They originated from different historical expansion paths of the breeding ranges, while the birds remained faithful to their wintering grounds, which might have been refuge areas during glaciation periods. Hence, the migration routes retraced the expansion paths of the breeding range and might have been constrained from shifting in some species by phylogenetic restrictions (Bensch et al., 2023). The migration direction of largely solitary migrating songbirds nonetheless sometimes undergoes selection through adaptation processes (Justen & Delmore, 2022; Liedvogel et al., 2011).

From early studies on captive Eurasian Blackcaps, it has been hypothesized that the direction of migration is controlled by a few genes (Helbig, 1991). Research on revealing the genetic basis of migration direction has been carried out in several songbird species that have a migratory divide (Bascón-Cardozo et al., 2024 ; Bensch et al., 1999, 2009; Delmore et al., 2020a; Helbig, 1996; Lundberg et al., 2017; Ruegg et al., 2002, 2014; Sokolovskis et al., 2023; Turbek et al., 2022; Zhao et al., 2020). Interestingly, the genetic signals associated with phenotypic variation detected hitherto typically differ between study systems (Caballero-López & Bensch, 2024). Parallel evolutionary histories and differential environmental factors along different flyways may have thus promoted convergent evolution (Bensch et al., 2023; Caballero-López & Bensch, 2024).

Conspecific phenotypic variation in migration patterns can in turn also facilitate the speciation process by acting as barriers for gene flow. Helbig (1991) proposed that in Eurasian Blackcaps, crossbred offspring from parental individuals migrating to Southeast and Southwest in autumn would follow an intermediate route south across the Alps and the Mediterranean Sea. Assuming that this route was suboptimal and associated with high fatality, it was hypothesized to function as a mechanism of post-zygotic reproductive isolation limiting the gene flow between populations (Helbig, 1991). However, since then viable intermediate routes have been demonstrated to exist in this system (Delmore et al., 2020b, 2023). Alternatively, or in addition to possible differentiation due to spatial factors, temporal factors can restrict gene flow (Bearhop et al., 2005; Turbek et al., 2022). For example, allochrony in spring arrival phenology can be a pre-zygotic reproductive isolation mechanism, resulting in a trend of assortative mating between parents with similar arrival times (Bearhop et al., 2005; Taylor & Friesen, 2017; Turbek et al. 2022; Veen et al., 2001, 2007).

While major advances have been achieved in studying the evolution of migration traits, Asian landbird species have rarely been taken into consideration (Irwin et al., 2005b; McKinnon & Love, 2018; Turbek et al., 2022). In Asia, the QTP and its surrounding mountains and deserts potentially present a significant barrier to migration for species breeding in Siberia, within the range of the QTP, and wintering south of the QTP (Zhao et al., 2024). With an average elevation over 4,000 m above sea level (a.s.l.) and an area over 2.5 million km^2^, crossing the massive plateau demands efficient respiration rate and usage of oxygen, tolerance of changeable weather, and endurance, given large barren areas with restricted refueling opportunities (Hawkes et al., 2011; Kumar et al., 2020). Species that are known to migrate across it at high altitude, e.g., the Bar-headed Goose (*Anser indicus*), the demoiselle crane (*Grus virgo*), and the Brown-headed Gull (*Chroicocephalus brunnicephalus*), exhibit special behavioral and physiological adaptations (Bishop et al., 2015; Galtbalt et al., 2022; Mi et al., 2022; Prins & Namgail, 2017; Yu et al., 2024). Although rarely discussed, several small highland-specialized songbirds are believed to also migrate across the QTP: White-throated Bushchat (*Saxicola insignis*) occasionally occurs on the QTP during the spring migration season (Collar, 2020; Urquhart, 2010), and Siberian Stonechats and Rosy Pipits (*Anthus roseatus*) have been documented to fly across the Himalayas down to Bhutan (Proud, 1949; Spierenburg, 2005). Otherwise, the majority of Siberian breeding passerine species and those breeding on the QTP migrate along detoured routes either along the eastern or western side of the QTP (Irwin et al., 2005b; Kumar et al., 2020; Zhao et al., 2024).

Here, we introduce the common stonechat complex (*Saxicola* sp.) in Asia as a novel system for studying the evolution of migratory birds facing the QTP barrier. The common stonechat complex occurs in Africa, Europe, and Asia, and shows a wide range of migratory phenotypes across its range (Justen et al., 2022; Urquhart, 2010).

**Figure 1.**
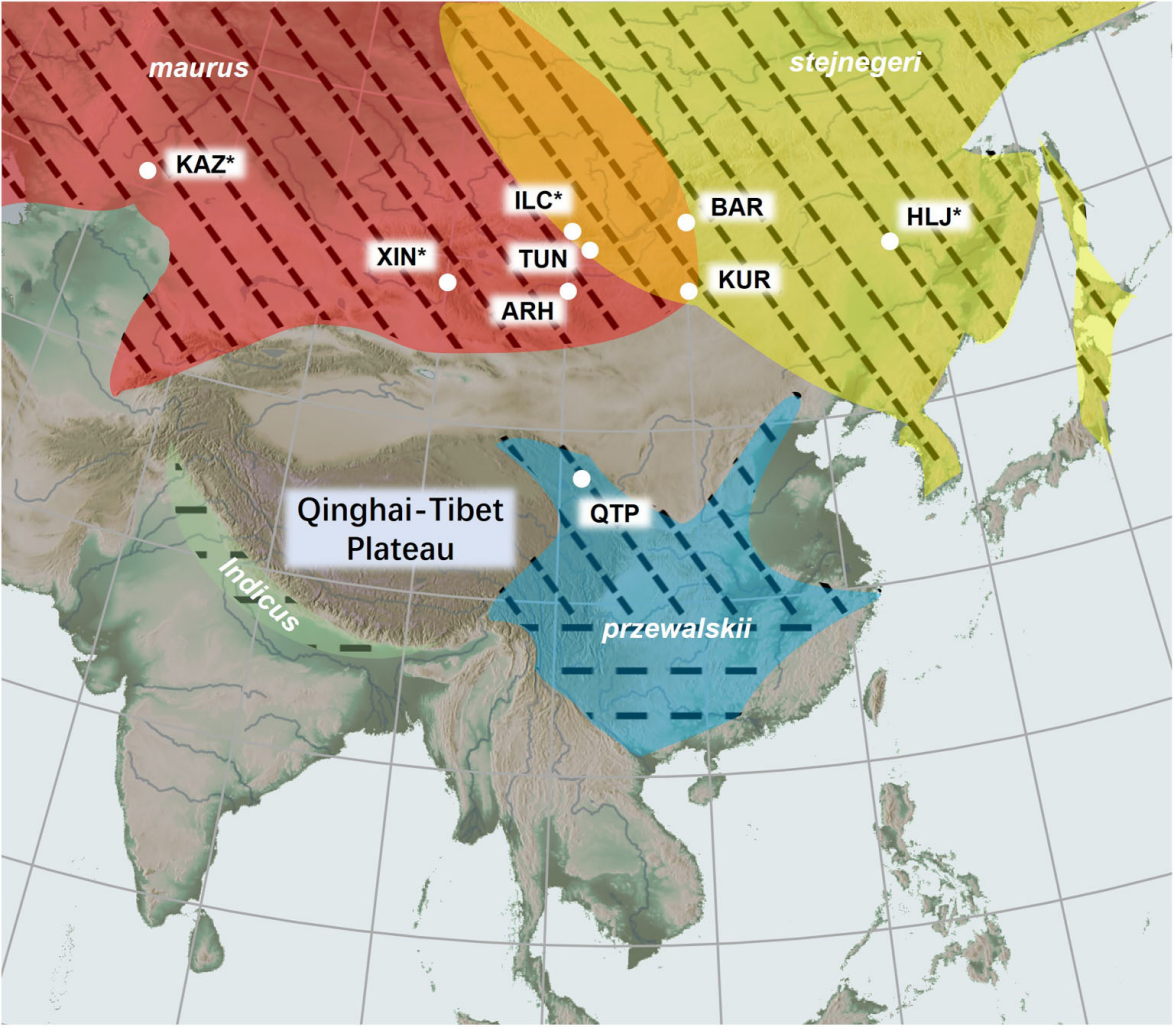
Schematic distribution map of stonechat taxa breeding in Asia (based on citizen science data from eBird.org and www.birdreport.cn, Opaev et al., 2018, and IUCN Red List distribution data). Four taxa are displayed on the map: *maurus* (red), *stejnegeri* (yellow), *przewalskii* (blue), and *indicus* (green); the presumably sedentary *indicus* was not included in this study. The overlapped area between *maurus* and *stejnegeri* represents the location of the hypothesized contact zone between the two taxa. The migration status is demonstrated by slashed dashed lines (migratory) and horizontal dashed lines (presumably partial or non-migratory, status unclear): *maurus* and *stejnegeri* are full migratory; *przewalskii* in the northern breeding range are full migratory, while the southern breeding range may be partial or non-migratory; *indicus* is assumed to be non-migratory but lacks information. The white dots represent the sampling sites involved in this study: Kazakhstan (KAZ), Xinjiang, China (XIN), Ilchir lake, Russia (ILC); Tunka valley, Russia (TUN), Barguzin valley, Russia (BAR), Arhangai valley, Mongolia (ARH), Khurkh, Mongolia (KUR), Qinghai, China (QTP), and Heilongjiang, China (HLJ). Location names with asterisks indicate that only genomic data were collected at these sites, and no individual migration tracking with geolocators was conducted.

High diversity of the migration phenotype also appears to exist among Asian stonechats (Figure 1). The nominate subspecies of Siberian Stonechat *S. m. maurus* and Amur Stonechat *S. stejnegeri* potentially share a migratory divide in central Siberia, ranging south down to Lake Baikal and potentially further into Mongolia (Irwin et al., 2005b). A tracking study of the Hokkaido (Japan) population of Amur stonechat revealed that they migrate through eastern China (Yamaura et al., 2017, 2026). Moreover, Tibetan Stonechat *S. przewalskii* breeding on the QTP and central to southwest Chinese mountain regions are believed to be migratory, but their migration patterns remain unclear (Urquhart, 2010). In contrast, Indian Stonechats (*S. m. indicus*) breeding in northeast India to Pakistan are believed to be sedentary (Urquhart, 2010). The ambiguous differentiation on color morphs between Siberian and Amur Stonechat (Hellström & Norevik, 2014; Hellström & Waern, 2023) complicates identifying them during the non-breeding season and further obscures their migration routes. Genetic studies on the stonechat taxa in Asia mainly conducted phylogenetic analyses on mitochondrial DNA and behaviors and plumages (Illera et al., 2008; Opaev et al., 2018; Zink et al., 2009), and did not associate migration routes with population genetic structural differences (Justen et al., 2022; van Doren et al., 2017a). The taxonomic status of stonechats is still poorly resolved (Drovetski et al., 2004; Illera et al., 2008; Opaev et al., 2018; van Doren et al., 2017a; Zink et al., 2009). To simplify the taxonomical reference in this publication, we will use *maurus, stejnegeri*, and *przewalskii* to refer to these stonechat taxa.

Taking advantage of the diverse migration behaviors of stonechats in Asia, we investigated the phenotypes and genotypes of migrants faced with the QTP. We focused on the two taxa breeding north of the QTP near the migratory divide (*maurus* and *stejnegeri*), and compared them to the taxon breeding on the QTP (*przewalskii*). For genomic analyses, we added samples from additional populations (Figure 1). Our objectives are (1) to document migratory phenotypic diversity using individual tracking methods and (2) to relate the migratory phenotypic variation to genetic diversity by employing whole genome re-sequencing. We aim to test the following hypotheses: (i) There is a migratory divide between *maurus* and *stejnegeri* in its documented contact zone in Siberia, and possibly in Mongolia, and migrants from either side will detour to avoid crossing the QTP; (ii) hybrids are rare, and if detected, employ an intermediate route compared to their parental phenotype; (iii) the highland breeding population *przewalskii* will also avoid crossing the QTP during migration; and (iv) there is clear genomic differentiation among *maurus, stejnegeri*, and *przewalskii*, associated with their migration patterns.

## Methods

### Fieldwork

Fieldwork was carried out at six different sites, five of which (excluding Ilchir) included individual tracking of adult birds (Figure 1). For the contact zone between *maurus* and *stejnegeri*, we selected three field sites west of the divide, i.e., in the Tunka Valley, Russia (102.33–102.39E, 51.74–51.81N, 800 m a.s.l.), at Ilchir Lake, Russia (100.9E, 51.9N, 1,900 m a.s.l.) and in a valley located in the Arhangai Province, Mongolia (100.86–100.91E, 48.72–48.74N, 1,900 m a.s.l.); and in two valleys east of the divide, i.e., Barguzin, Russia (109.79–109.82E, 53.56–53.58N, 500 m a.s.l.) and Khurkh, Mongolia (110.10–110.21E, 48.43–48.46N, 1,400 m a.s.l.). For *przewalskii*, the field site was in Menyuan Hui Autonomous County in Qinghai, China (101.30–101.54E, 36.64–37.43N, 3,300 m a.s.l.).

We conducted our fieldwork from 2020 to 2024 between late May and early July during the breeding season of the local stonechat populations. We first scouted the area to identify nest sites, and then applied a combination of mist nets, perch traps, and ground bait traps near the nests to catch adult breeders. We equipped each individual with one type of light-level geolocators (specifics are introduced in the next section), and banded the individual with an aluminum ring and an individual-specific combination of color rings. The aluminum rings were provided by the National Bird Banding Centre of China, the Wildlife Science and Conservation Center of Mongolia, and the Bird Ringing Centre of Russia. After the handling, we released the birds back to their territories; the same individuals were spotted and captured by the same method to retrieve the loggers in later years if they returned. We also sampled blood from the brachial vein under the left wing for most of the captured individuals and stored it in 70% ethanol for DNA extraction.

### Geolocator deployment and analysis

We used light-level geolocators provided by two manufacturers: the Intigeo-P30Z11-DIP-NOT light-level recorder from Migrate Technology Ltd. (MT, 0.36 g [excluding harness], 14 × 6 × 3 mm, 11 mm light stalk); and the SOI-GDL2 logger from Swiss Ornithological Institute (SOI, 0.68 g [excluding harness], 8 × 6 × 4 mm 7 mm light stalk). Both logger types were programmed with the same settings to store light levels every 5 min from July 15 in the deployment year for as long as the battery lasted.

We deployed 115 geolocators (60 MT, 55 SOI) on 58 males and 57 females at five field sites between 2021 and 2023, and successfully retrieved 17 devices in subsequent seasons. Of those, 16 included complete data, whereas one logger that stopped working before the autumn migration and was excluded (Table 1). One logger provided data for two autumn and one spring seasons (CA903). The return rate of birds tagged with MT loggers (18.3%) was slightly higher than those tagged with SOI loggers (12.7%). The SOI logger data were extracted by the manufacturer; the MT logger data were extracted using the IntigeoIF interface and software (Migrate Technology Ltd.).

**Table 1.** Light-level geolocator deployment and re-capture from different field sites.

				Deployment number		Recollection number	
Country	Location	Deployment year	Logger type	Male	Female	Male	Female
Russia	Tunka (TUN)	2021	MT^a^	14	6	3	1
	Barguzin (BAR)	2021	MT	10	10	1	1
China	Qinghai (QTP)	2021	MT	9	11	1	4^c^
		2022	SOI^b^	7	8	1	1
Mongolia	Khurkh(KUR)	2023	SOI	8	12	0	1
	Arhangai(ARH)	2023	SOI	10	10	2	1 + 1^d^

a MT stands for Migrate Technology Ltd.

b SOI stands for Swiss Ornithological Institute.

c One logger (CA903) was deployed in 2021 and re-collected in 2023.

d One female lost its logger upon re-capture; the other female carried a logger (29EN) that stopped recording data before the autumn migration.

We conducted the analysis in R 4.3.2 ( R Core Team, 2024 ), following the manual for geolocator analysis (Lisovski et al., 2020). We used the SGAT package to analyze movement trajectories following the instructions on https://geolocationmanual.vogelwarte.ch/SGAT.html (Lisovski et al., 2020). Analysis details can be found in Supplementary methods and Table S1. To acquire robust locations for stopovers from the movement model, which are essential for confirming the route choices, we assumed a Gamma distribution of speed with *k* = 2.2 and λ = 0.8. This allowed the movement to be steadily continuous but detected fewer stationary sites, prioritizing robustness over number of stopovers.

### Whole-genome sequencing

To investigate the population genetic structures among Asian migratory populations, especially in the contact zone and the Qinghai population, we selected 77 samples from seven geographical breeding populations (Figure 1; Table S2). No genomic samples were available from the Mongolian study sites (ARH, KUR). To strengthen population genetic analyses, we complemented samples from our field sites with historical samples from captive Kazakh birds (Justen et al., 2022) and from Xinjiang, China, presumably representing *maurus*, and from Heilongjiang, China, presumably representing *stejnegeri*. Both Xinjiang and Heilongjiang samples were specimen samples archived in the Institute of Zoology, Chinese Academy of Science. Sample sizes are as follows: Kazakhstan (KAZ, *n* = 9), Xinjiang, China (XIN, *n* = 4), Ilchir Lake, Russia (ILC, *n* = 2), Tunka Valley, Russia (TUN, *n* = 18), Barguzin Valley (BAR, *n* = 17), Heilongjiang, China (HLJ, *n* = 4), and Qinghai, China (QTP, *n* = 23) (Table S2). Of the 16 birds whose tracking data are included, we had genomic information for 11 individuals (TUN_09, TUN_10, TUN_16, TUN_17, BAR_05, BAR_10, QTP_04, QTP_07, QTP_08, QTP_09, and QTP_18, Table 2; Table S2).

**Table 2.** Basic information of each tracked individual and their migration spatio-temporal patterns.

ID	Genomic sample ID	Origin	Sex	Age	Route type	Year	Autumn departure	Autumn arrival	Autumn duration (day)	Spring departure	Spring arrival	Spring duration (day)	Bee-line distance (km)	Autumn migration distance (km)^a^	Spring migration distance (km)^a^	Prolonged autumn distance (km)	Prolonged spring distance (km)
CA863	BAR_10	Barguzin	F	2	East	2021	08–15	10–01	47	04–20	05–24	34	3,880	4,082	4,490	202	610
29AM		Khurkh	F	2+	Intermediate	2023	08–29	09–29	31	04–13	05–04	21	3,437	3,692	5,038	255	1,601
CA869	TUN_10	Tunka	M	3+	Intermediate	2021	09–04	10–12	38	04–08	05–06	28	3,163		3,461		298
CA886	TUN_09	Tunka	M	2+	Intermediate	2021	08–28	09–23	26	04–09	05–10	31	3,700	4,279	4,854	579	1,154
CA892	BAR_05	Barguzin	M	3+	Intermediate	2021	08–30	09–30	31	04–07	05–17	40	4,024	4,418	4,834	394	810
CA865	TUN_17	Tunka	M	2	West	2021	08–18	09–29	42	04–20	05–14	24	3,362	4,843		1,481	
CA874	TUN_16	Tunka	F	2+	West	2021	08–22	09–30	39	04–27	05–19	22	3,363	4,444	4,056	1,081	693
27XK		Arhangai	M	2	West	2023	08–31	10–08	38	04–21	05–15	24	3,424	5,384	4,907	1,960	1,483
29AI		Arhangai	M	2	West	2023	08–29	10–14	46	04–19	05–14	25	3,021	4,833	4,455	1,812	1,434
CA903	QTP_07	Qinghai	F	3+	Highland	2021	09–11	10–28	47	03–27	05–02	36	2,032	2,216	2,536	184	504
CA903	QTP_07	Qinghai	F	3+	Highland	2022	09–11	10–14	33								
CA905	QTP_18	Qinghai	F	2+	Highland	2021	09–08	10–01	23	03–15	05–01	47	1,853		2,691		838
CA915	QTP_04	Qinghai	F	3+	Highland	2021	09–11	10–23	42	04–13	05–08	25	1,686	2,297	2,851	611	1,165
CA917	QTP_08	Qinghai	F	3+	Highland	2021	09–13	10–14	31	03–13	04–30	48	1,779				
CA921	QTP_09	Qinghai	M	3+	Highland	2021	09–07	10–19	42	03–13	04–20	38	1,768		1,899		131
29EH		Qinghai	M	3+	Highland	2022	09–12	10–15	33	03–25	04–24	30	1,991				
29AN		Qinghai	F	2+	Highland	2022	09–09	10–09	30	03–22	04–21	30	1,952	2,009	2,482	57	530

a The migration distance was calculated only when at least one stopover was detected in the SGAT analysis.

We extracted DNA with the Qiagen DNeasy Blood & Tissue Kits. The QTP samples were sequenced at a mean depth of 15X at Berry Genomics Inc., on an Illumina Novaseq instrument using a PCR method for pair-end reads of 150 bp. The remaining samples were sequenced at a mean depth of 15–20X at BGI Genomics Inc. for BGI DNBseq (WGS) using a PCR method for pair-end reads of 100 or 150 bp.

To identify single-nucleotide variants, WGS reads of both platforms were processed with the *variant-calling* pipeline, a custom-built *snakemake* workflow (gmanthey/variant-calling v1.0; https://doi.org/10.5281/zenodo.15969023; Mölder et al., 2021). Detailed descriptions can be found in Supplementary methods (Chen, 2023; Chen et al. 2018; Danecek et al. 2021; Vasimuddin et al. 2019). The reference genome we used for mapping and variant calling was assembled by van Doren et al., 2017a (GCA_900205225.1 on GenBank).

To investigate the population genetic structures and explore the hybridization level at the contact zone between *maurus* and *stejnegeri*, we used the SNP datasets for several population genetic analyses. We further ran a filter process in Plink 1.07 (Purcell et al., 2007), based on (1) Minor Allele Frequency (MAF) > 0.05 and (2) SNPs in LD filtered with a window size of 50 SNPs (advancing 10 SNPs at a time) and an *r*^2^ threshold of 0.2. We also excluded individuals KAZ_02 and KAZ_06 as they were found to originate from the same broods as KAZ_01 and KAZ_05, respectively. Lastly, we excluded QTP_06 and QTP_07 after a kinship test. We did not remove TUN_12 or TUN_14 even though they had close kinship, because they were from the contact zone.

To visualize the population structure and identify hybrid individuals, we used the Admixture software (Alexander et al., 2009) to run admixture analyses from a range of *K* = 1–5, with a biological hypothesis of *K* = 3 (*maurus, stejnegeri*, and *przewalskii*). We ran the analyses with 30 iterations, and picked the output with lowest Cross-Validation (CV) error for each *K* from all iterations. We also ran PCAs on the same SNP dataset using the distance-matrix function in Plink; the first test used all individuals, whereas the second test excluded the QTP individuals. For both tests, we extracted the PC1 and PC2 to visualize population structure. We did additionally, to determine the type of the hybrids in the contact zone (Barguzin, Tunka, and Ilchir) between *maurus* and *stejnegeri*; we used the triangular package (Wiens, 2025) and selected 647 diagnostic SNP loci (difference threshold at 0.9) using KAZ+XIN as P1 (*maurus*) and HLJ as P2 (*stejnegeri*) to illustrate the hybrid index and interclass heterozygosity pattern among individuals from the contact zone. All visualization was conducted in R 4.3.3 (R Core Team, 2024).

## Results

### Migration routes

**Figure 2.**
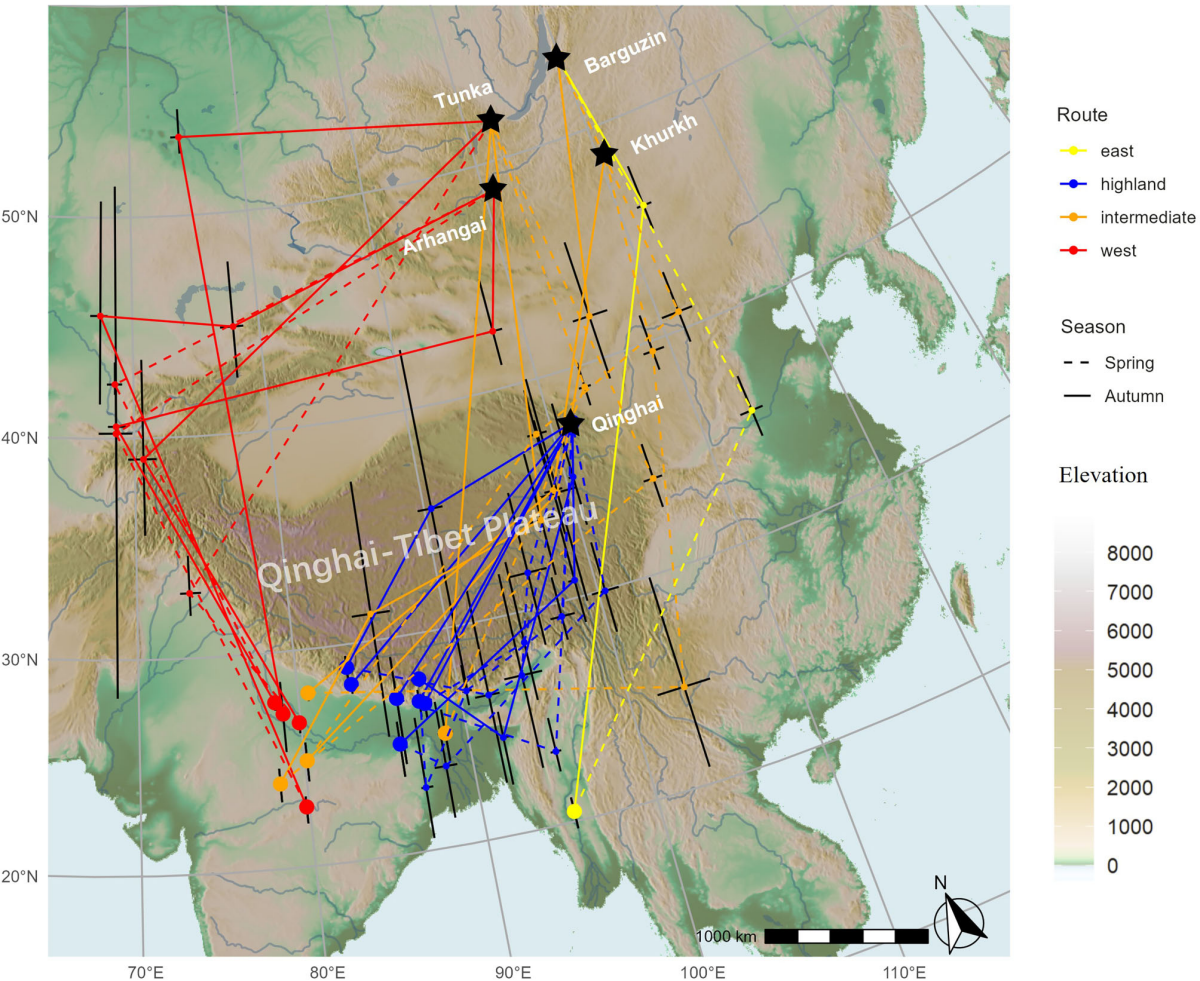
Migration routes of stonechats from different populations in Asia, estimated based on light-level geolocator data. The five deployment locations (breeding sites) are marked with names and black stars. The Qinghai-Tibet Plateau (QTP) is labeled. Four types of migration routes are subjectively classified and displayed in different colors. Wintering locations are displayed as large dots, whereas stopover sites are displayed as small dots. The center of the dot represents the median location estimated from the Markov chain Monte Carlo (MCMC) model in the SGAT analysis, whereas the error bars are drawn according to the 2.5% and 97.5% of the latitude/longitude estimation from the MCMC model. The color of the background map refers to the ground elevation. Potential autumn routes are drawn as solid lines, whereas potential spring routes are drawn as dashed lines. Routes are estimated as the shortest trajectory between detected stationary sites.

We acquired 17 autumn and 16 spring migration tracks of 16 individuals (8 males and 8 females) (Figure 2; Figure S1). Of the four individuals recaptured from the Tunka population, west of Lake Baikal, two individuals followed the western route in both autumn and spring, and wintered in north India; the other two followed an intermediate route in both seasons, where one wintered in central India and the other wintered on the south side of the east range of the Himalayas. Of the two individuals recaptured from the Barguzin population east of Lake Baikal, one followed the eastern route in both seasons and wintered in the lowland region in central Myanmar; the other followed an intermediate route and wintered in central India. The two individuals recaptured from the western Mongolian Arhangai population both followed the western route in both seasons, and wintered in north India. The individual recaptured from the eastern Mongolian Khurkh population followed an intermediate route during autumn and the eastern route during spring, and wintered on the south side of the middle range of the Himalayas. All seven individuals recaptured from the Qinghai population migrated across the QTP in autumn and spring, and wintered around the eastern range of the Himalayas. The data precision did not allow us to judge whether these individuals wintered on or off the QTP; however, four out of seven individuals had one or several detected stopover(s) around the Assam Plain during spring, which is connected with the QTP through the Yarlung Zangbo Grand Canyon.

Relative to the bee-line distance, the migration distances of both seasons for each individual are shown in Table 2. The migration distance of the Tunka individuals was 1,281 ± 200 km (38%) longer than the bee-line distance, and of the Arhangai individuals, it was 1,886 ± 74 km (59%) longer. For the individual following the eastern route from Barguzin, their migration distance was 610 km (16%) longer. We calculated the autumn and spring migration distance separately for the individuals following an intermediate route, as their routes were not always consistent between seasons: in autumn, the migration distance exceeded the bee-line distance by 409 ± 133 km (11%), and in spring, by 966 ± 477 km (27%) (Table 2 and Figure 2; Figure S2).

### Migration phenology

**Figure 3.**
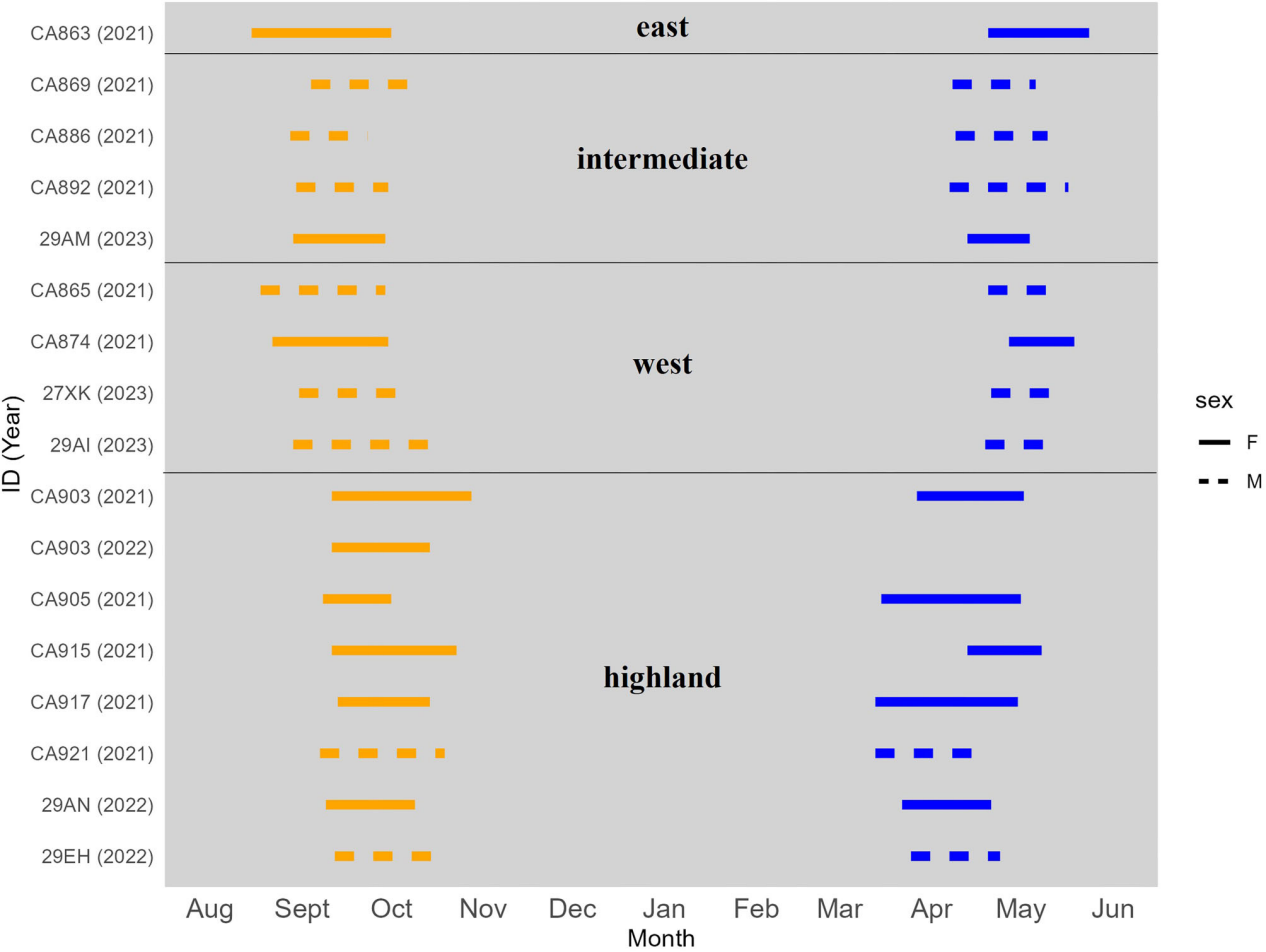
Individual migration schedules grouped by four types of migration routes (see Figure 2). The bars represent the duration of the migration; autumn migrations are in orange, whereas spring migrations are in blue. Females are displayed with solid lines, whereas males are displayed with dashed lines.

Autumn migration occurred between late August to early October in the Russian and Mongolian populations, with an average duration of 38 ± 7 days, while spring migration occurred between early April and mid-May in these populations, with an average duration of 28 ± 6 days. In the Chinese population (Qinghai, also as the highland route category), autumn migration occurred between early September and late October, with an average duration of 35 ± 7 days; and spring migration occurred between mid-March and early May, with an average duration of 36 ± 8 days (Figure 3 and Table 2).

### Population structure and hybridization level inferred by WGS data

We acquired 18,837,382 SNPs after the extensive filtering after the variant calling step, and 594,881 SNPs were retained after the second round of filtering, with a genotyping rate at 0.995. One individual from the QTP (QTP_11) population and two from XIN (XIN_02, 03) were filtered out due to low data quality.

**Figure 4.**
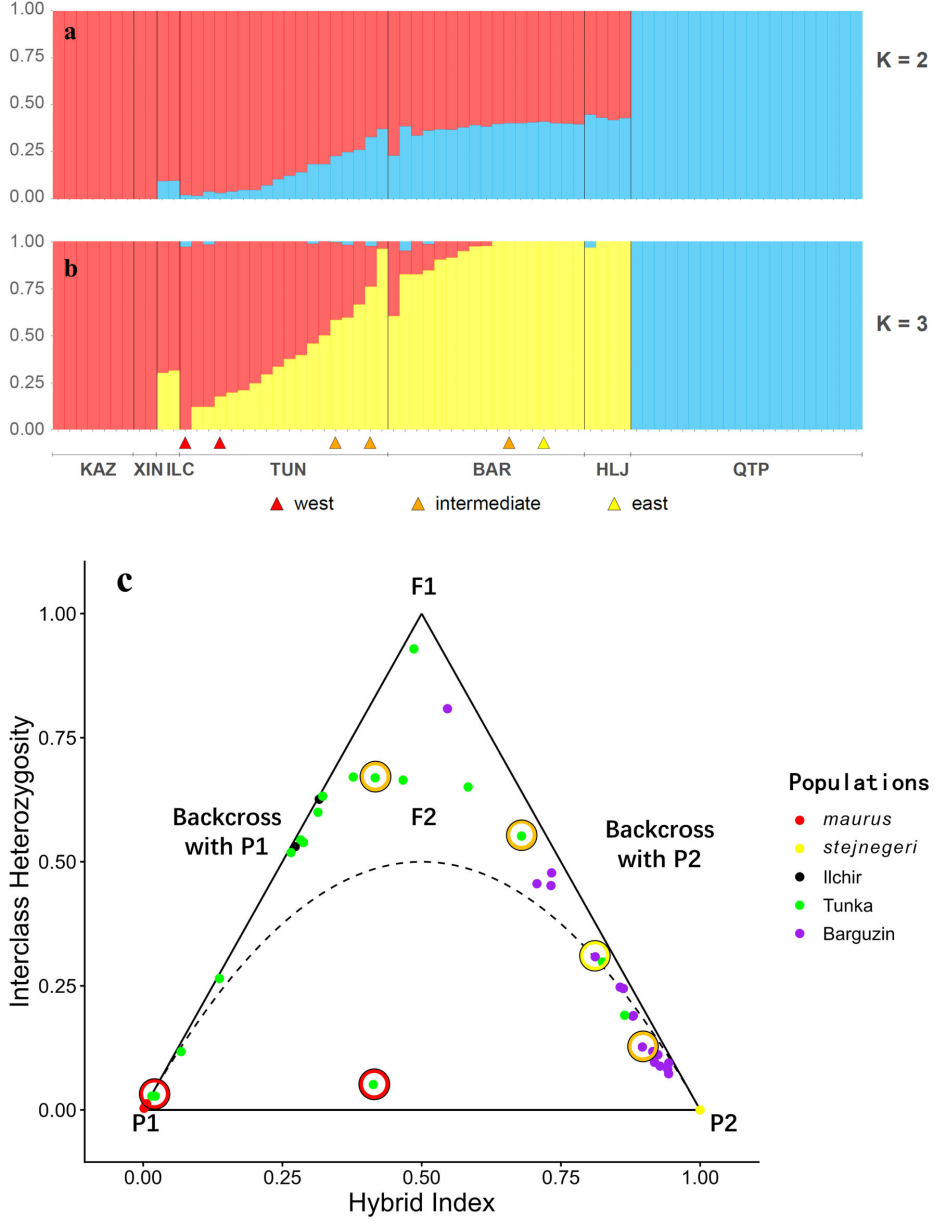
**a** and **b**) Admixture plots (*K* = 2 and 3) of individuals from Kazakhstan, Xinjiang, Ilchir, Tunka, Barguzin, Heilongjiang, and Qinghai, based on the whole-genome re-sequenced data after filtering. Each individual follows the same order in each plot. Black vertical solid lines separate individuals from different populations, with a reference to the population along the x-axis at the bottom of the plot. The yellow, orange, and red triangles under the corresponding bars refer to the individuals with tracking data, and the colors refer to the route groups: red represents the western route, orange represents the intermediate route, and yellow represents the eastern route. (**c**) The triangle plot of the same group of individuals as included in the admixture plots, except for those from Qinghai. The plot is defined by solid lines: *y* = 2*x* and *y* = −2*x* + 2, and dotted curve *y* = 2*(1 − *x*). The dotted curve represents the boundary below which individuals cannot occur, assuming Hardy–Weinberg equilibrium. For parental groups, P1 were set as *maurus* using KAZ+XIN individuals, P2 as *stejnegeri* using HLJ individuals. The theoretical positions of different types of hybrids (F1, F2, backcross with P1, and backcross with P2) were labeled on the triangle plot. Individuals with tracking data are circled, following the same color code as described in panels a and b.

In the admixture analyses (Figure 4), the *K* = 2 model (Figure 4a) held the lowest CV error value (0.5028 ± 0.0004), where birds from the QTP (*przewalskii*) and from the westernmost KAZ+XIN (*maurus*) populations were categorized as two populations, respectively. All others were identified as their hybrids with different hybridization levels. The easternmost HLJ population (*stejnegeri*) had a genetic ancestry of 40%–44% from the QTP population in this model. Individuals from the assumed Siberian contact zone in the Lake Baikal region, ILC, TUN, and BAR, carried 1%–39.9% ancestry from the QTP population. For the *K* = 3 model (Figure 4b), which was our primary hypothesis according to the presumed taxonomy (*maurus, stejnegeri*, and *przewalskii*), the CV error value (0.5241 ± 0.0006) was slightly higher than for *K* = 1 (0.5081 ± 0.0002) or *K* = 2, but clearly lower than for *K* = 4 (0.5778 ± 0.0049) or 5 (0.6330 ± 0.0082) (Figure S3). In this model, QTP (*przewalskii*) and the westernmost KAZ+XIN (*maurus*) populations were again categorized as two populations, whereas the easternmost HLJ (*stejnegeri*) population was categorized as the third population. In the *K* = 3 model, 8 of 17 individuals from the BAR population from the eastern Baikal region had complete *stejnegeri* genetic composition. The remaining birds carried a 2.5%–40% genetic ancestry of *maurus*. In the western Baikal ILC+TUN populations, no birds showed the expected complete *maurus* ancestry. For 5 of 20 individuals, the genetic ancestry of *maurus* was below 50% (4%–41%), for another nine individuals it ranged between 50% and 75%, and for the remaining six individuals, ancestry of *maurus* was above 75% (79%–97%). All but one individual (TUN_17) had some genetic ancestry of *stejnegeri* (12%–96%).

To zoom into the contact zone between *maurus* and *stejnegeri*, the genetic composition of individuals was presented in a triangle plot. The parental group P1 was set as *maurus* using KAZ+XIN individuals, and the parental group P2 was set as *stejnegeri* using HLJ individuals (Figure 4c). The two individuals from Ilchir shared a pattern similar to a backcross with P1. Of the 17 individuals from Barguzin, 16 positioned between a pure P2 and a backcross with P2 on the plot; the remaining individual positioned close to an F1. Among the 18 Tunka individuals, one positioned close to an F1, three positioned close to a backcross with P2, nine positioned between a pure P1 and a backcross with P1 and four positioned close to be an F2; the remaining individual could not be categorized due to an intermediate hybrid index combined with low interclass heterozygosity.

**Figure 5.**
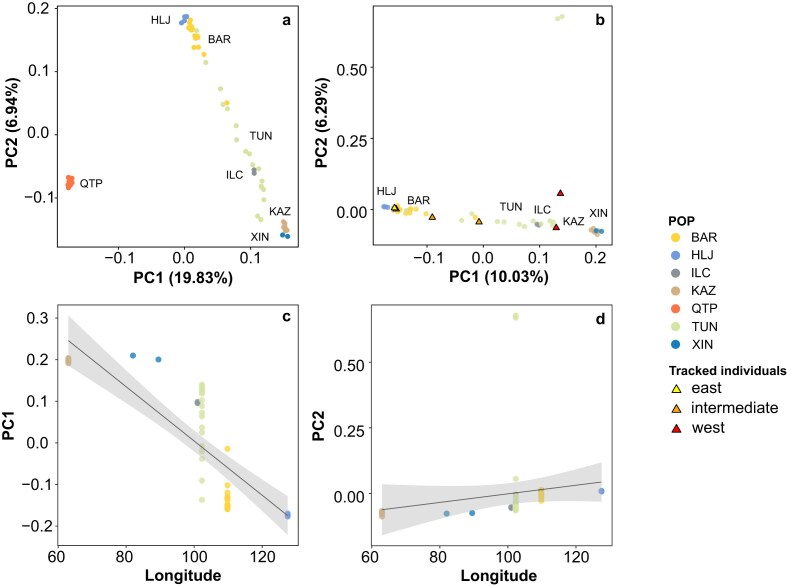
(a) PCA plot (PC1 ~ PC2) of individuals from Kazakhstan, Xinjiang, Ilchir, Tunka, Barguzin, Heilongjiang, and Qinghai, based on the whole-genome re-sequenced data after filtering. The percentage of variation explained by each PC is indicated in parentheses, and samples are colored by population. (b) PCA plot (PC1 ~ PC2) from the same set of individuals but the Qinghai population. For the six birds with both phenotypic and genotypic data from the contact zone, dots were marked by triangles, with a color code referring to the route groups: red represents western route, orange represents intermediate route, and yellow represents eastern route. Please note that one orange triangle largely overlapped with the yellow one. (c and d) The relationship between PC1 (c) and PC2 (d) with breeding longitudes of all sampled individuals but the Qinghai population. The colors of the dots refer to the same population color code as in panels a and b. The regression lines show standard deviation.

In the first PCA including all individuals (PC1~PC2, Figure 5a), the samples from the QTP population were clearly clustered and separated from the rest of the samples. All other birds in the dataset were linearly distributed along a diagonal line between PC1 and PC2, ranging from the easternmost HLJ to the westernmost KAZ+XIN populations. The second PCA excluding the QTP individuals showed similar linear patterns along PC1 from the easternmost HLJ to the westernmost KAZ+XIN populations (Figure 5b); there was a significant linear correlation between sampling longitude and PC1 (adjusted *R*^2^ = 0.62, *p* < 10^−12^; Figure 5c). The variation on PC2 was marginal except for TUN_12 and TUN_14, which grouped separately from the other individuals on PC2 (Figure 5b) and shared high kinship. The correlation between sampling longitude and PC2 was also significant after excluding TUN_12 and TUN_14 (adjusted *R*^2^ = 0.59, *p* < 10^−12^; Figure 5d).

### Relationship between migration route diversity and genetic variation

For the two individuals following the western route (western Baikal region TUN_16 and TUN_17), genetic components for *maurus* were 75% and 97% in the *K* = 3 admixture model (Figure 4b), and they were located very close to the westernmost KAZ+XIN populations’ cluster in the PCA plot (Figure 5b). They both had very low interclass heterozygosity level, despite the hybrid index of one of them being close to 0.5 (Figure 4c). The Barguzin individual following the eastern route was inferred to be a pure *stejnegeri* in the *K* = 3 admixture model (Figure 4b), and it clustered with the easternmost HLJ individuals in the PCA plot (Figure 5b). This individual nonetheless positioned as a backcross type with *stejnegeri* in the triangle plot with an interclass heterozygosity near 0.25 (Figure 4c). The three individuals from Barguzin or Tunka, which followed intermediate routes, showed 60%–95% genetic contributions of *stejnegeri* in the *K* = 3 admixture model (Figure 4b). These three individuals were all located between the western-routed and eastern-routed individuals in the PCA plot, but were closer to the HLJ cluster (Figure 5b). Two of the intermediate-routed individuals positioned in the range of F2 in the triangle plot, among which one was closer to a backcross with *maurus* while the other closer to a backcross with *stejnegeri*. Surprisingly, the third individual was positioned very close to the parental *stejnegeri* type with lower interclass heterozygosity score than the east-route individual (Figure 4c).

## Discussion

In this study, we used individual tracking data to acquire migration route phenotypes from several populations of stonechats in Asia. We confirmed the hypothesis that there is a migratory divide of stonechats in the contact zone between *maurus* and *stejnegeri* south of Lake Baikal (Irwin et al., 2005b) and showed that the divide extended to eastern Mongolia. We rejected the hypothesis that the *przewalskii* population breeding on the QTP would also detour from the QTP via the lowland region in central China, as recently described for the sympatric breeding population of Siberian rubythroat (*Calliope calliope*) (Zhao et al., 2024). Instead, they migrated exclusively over the highlands during autumn migration. Using the combined dataset from tracking and genomic resequencing, we also rejected the hypothesis that hybrids between *maurus* and *stejnegeri* are rare and demonstrated extensive hybridization. The association between hybridization and the newly discovered intermediate route still remains unclear. Lastly, we demonstrated a clear population genomic structure among *maurus, stejnegeri*, and *przewalskii*, partly associating with population-specific migration route phenotypes.

### Routes relative to geographical barriers in central Asia

In our study, we revealed the various migration route choices among stonechats in Asia, with seemingly different responses to the presumed geographical barrier, the QTP.

The route around the north-western side of the QTP, i.e., the west route, has been documented in many bird species via tracking, e.g., the Demoiselle Crane (*Grus virgo*) (Galtbalt et al., 2022; Mi et al., 2022) and Siberian Bluethroat (Strubbe et al., 2025). We showed that this route is also being used by stonechats. This route encompasses a large detour of up to 59% compared to the bee-line distance (Table 2 and Figure 2; Figure S2). Benefits of barrier avoidance may be one explanation for this pattern. Through selection or energy limitation, the QTP, along with other geographical barriers such as the Taklamakan Desert or the Tian Shan mountains, may counter-act migration routes that directly cross these landscapes. Nonetheless, the documented western detour still passes unhospitable regions for the birds, such as the Tian Shan and Pamir mountains. In our data, we detected one stopover range in both seasons that was used multiple times (2/4 tracks in autumn and 2/4 tracks in spring), located around the Chirchiq River Valley, close to Tashkent, Uzbekistan (Figure 2; Figure S1). As an oasis along the route, it may function as a stopover refueling site and resting place where birds can wait for optimal weather and wind conditions before crossing unhospitable regions, or rest and refuel after barrier-crossing flights.

The route around the eastern side of QTP, i.e., the east route, found in a single individual, involved a smaller detour of around 16% from the bee-line distance (Table 2) and was linked to a more easterly wintering location. The tracking of Amur Stonechats (*stejnegeri*) from Japan suggested that they migrated through east to central China toward their wintering sites in Thailand (Yamaura et al., 2017, 2026); the similarity to the Japanese migration pattern further supported our documentation of the east route. This east route also resembles the previously documented migration path of Siberian Rubythroats (Zhao et al., 2024), and seemingly follows the mountain chain in central China. Located between the Central Asia Flyway and the East Asia Flyway, central China may be frequently used by species migrating on the east side of the QTP (Heim et al., 2018, 2020; Kumar et al., 2020; Zhao et al., 2024).

Between the western and eastern routes, the intermediate routes used by four stonechats from the contact zone between *maurus* and *stejnegeri* passed the eastern side of the QTP and therefore crossed considerable elevations of over 3,000 m a.s.l.. We speculate that this behavior is facilitated by stonechat taxa being adapted to high elevations, as they are known to breed at high elevation ranges in Asia and Africa (Collar, 2021).

Parallel to the routes from stonechats breeding in Siberia and Mongolia, we document for the first time a “highland route” used by the population breeding on the QTP, in accordance with our high-elevation adaptation hypothesis. The *przewalskii* stonechats are believed to breed both on QTP and along the lower-elevation mountains in central China, north up to Shanxi and Beijing, and south down to Yunnan (Figure 1); therefore, we had hypothesized that the QTP population would leave the highland, merging with the central Chinese populations during migration. However, none of the tracked individuals showed any signs of bypassing the QTP along its east side.

All tracked individuals, including those using intermediate routes, were re-captured as breeders. Although we acknowledge reporting bias because only surviving birds are recaptured, we thereby demonstrated viability of crossing the QTP in several individuals. Geographically, we did not document any migration trajectory crossing the western part of the QTP, where the land is more barren (Liu et al., 2017) and the elevation is higher with more mountains over 5,000 m a.s.l.. This asymmetry may suggest that for stonechats, the actual barrier for optimal migration is limited to the western part of the QTP. A greater barrier effect of the western QTP might also be linked to differences in vegetation and habitat availability: For example, it features more alpine deserts, but fewer alpine meadows than the eastern part (Dong et al., 2020).

Our data stand out as providing the first tracking evidence of small songbirds crossing the QTP. Based on the known ability of stonechats to inhabit high elevations, we suggest that the level of the barrier effect from this region may be taxon-specific, supported by possibly underestimated migration routes along the eastern part of the QTP of other high-elevation breeding songbird species, e.g., the White-throated Bushchat, Blyth’s Pipit (*Anthus godlewskii*) and Rosy Pipit (Collar, 2020; Tyler & de Juana, 2020; Tyler et al., 2020). Given the complexity of the landscape patterns and their differential interaction with different species, we propose that a careful revisit of the actual and region-specific barrier effect of the QTP and the surrounding landscapes should be undertaken, to understand how the landscapes in Asia have shaped the diversity of avian migration.

The diverse migration patterns we described for stonechats may be paralleled by other species that are suggested to have a Siberian migratory divide but also share breeding populations on the QTP, for instance Citrine Wagtail, White Wagtail (*Motacilla alba*), and Greenish Warbler (*Phylloscopus trochiloides*) (Irwin et al., 2005b). Migration patterns in these species may resemble the pattern in stonechats, where both highland routes and detoured routes around the QTP exist in parallel. We encourage further studies on different taxa to clarify the role of the QTP and surrounding barriers.

In this study, we have not been investigating the phenological patterns of migration in depth, yet there seem to be population-specific patterns (Figure 3). Because stonechat systems have been long used in studies of biological timekeeping (Gwinner & Dittami, 1990; Helm & Gwinner, 2006; Helm et al., 2009; Justen et al., 2022; van Doren et al., 2017b), we expect future studies using our data to reveal environmental and genetic mechanisms responsible for phenological differences in migration.

### Population structure, hybrid zone properties, and genetic perspective for the migration direction

From genomic resequencing, we infer frequent genetic exchange between *maurus* and *stejnegeri* in the contact zone. We demonstrated that most individuals from Ilchir and Tunka (19/20), and half of the individuals from Barguzin (9/17), had genetic ancestry of both *maurus* and *stejnegeri*, based on the admixture analysis (*K* = 3) (Figure 4b). Aside from *przewalskii* stonechats, there was a clear linear relationship between both PC1 and PC2 with the breeding longitude, from pure *maurus* (Kazakhstan and Xinjiang, China) to pure *stejnegeri* (Heilongjiang, China) populations (Figure 5c and d). Low *F*_ST_ along these Siberian populations also indicated low fixation level of population genetic features (Table S3). We therefore shift the terminology from “contact zone” to “hybrid zone.”

The hybridization between *maurus* and *stejnegeri* might be extensive. In the admixture plot (*K* = 3), the high frequency of individuals with around 25% and 75% genetic contributions of *maurus* or *stejnegeri* supported the existence of F2 hybrids and backcrosses (Figure 4b). In the triangle plot (Figure 4c), a substantial number of individuals were categorized as F2 and backcrosses, also suggesting prolonged and ongoing genetic mixing over many generations.

The likely continuous admixture may have contributed to the lower-than-expected number of predicted populations by the STRUCTURE model (Lawson et al., 2018). Additionally, if the sampled populations were actually derived from an ancestral population or lineage that was *not* sampled in our study, a so-called “ghost” population, this could also lead to an under-estimation of the number of populations (Lawson et al., 2018). To resolve the potential existence of a ghost population, we would require genomic sequences of a more extensive set of stonechat species, so that ancestry can be inferred in a more comprehensive phylogenetic context.

The violated expectation from Hardy–Weinberg equilibrium shown from the triangle plots by several individuals positioned below the dotted curve (Figure 4c) inviting further investigation (Wiens et al., 2025). Opaev et al. (2018) have shown differences in songs between *maurus* and *stejnegeri*, which indicated an assortative mating scenario. Anecdotally in the field, we observed that female CA874 (TUN_16, west route, arriving May 19) and male CA886 (TUN_07, intermediate route, arriving May 10) formed a breeding pair in 2022, after having been paired with other mates in 2021. Also based on the distribution of individuals in the triangle plot, we assume that route choices may not have strongly contributed to potential assortative mating mechanism. An expansion of data beyond the 647 diagnostic loci identified for the triangle plot may help us to understand the hybridization pattern over the genomic level, as some of these diagnostic loci might be linked.

Our results also enabled a better characterization of the hybrid zone than in previous studies using haplotyped mitochondrial DNA (Illera et al., 2008; Opaev et al., 2018; Zink et al., 2009). The relatively uniform “*stejnegeri*-dominant” genotype of stonechats at Barguzin, east of Lake Baikal (Figure 4), and its low *F*_ST_ score, compared with the easternmost Heilongjiang population, suggest that Barguzin is close to the border of the hybrid zone where the eastern genotype prevails. On the western side, our study sites in Tunka Valley and at Ilchir Lake are still within the hybrid zone, albeit with diminished contributions of *stejnegeri*. Local birds in this zone phenotypically resemble pure *maurus* (M. Hellström, personal observation). Therefore, the western border of the hybrid zone must be further extended to the west. In the absence of genomic data from Eastern Mongolia, the only cue to a possible extension of the hybrid zone between *maurus* to *stejnegeri* comes from an individual migrating along an intermediate route at Khurkh. Overall, our findings are disagreeing with Opaev et al.’s (2018) argument of an abrupt transition between *maurus* and *stejnegeri* in Transbaikalia. Due to the massive representation of intermediate genotypes, the identification by plumage and songs in the contact zone may not be an effective and accurate measure for species identification. More sequencing data and a cline analysis for phenotypic traits with genomic data may help to reveal the complex dynamics between various phenotypic differentiations and the hybridization background.

In our results, the relationship between intermediate route phenotype and hybrid genotype was not clear. On the one hand, the two intermediate route individuals from Tunka indeed carried a “*stejnegeri* dominated” hybrid genotype (Figure 4b), and both were categorized as F2 or backcross hybrids (Figure 4c). On the other hand, the intermediate route individual from Barguzin carried a full *stejnegeri* ancestry (Figure 4b) and in the triangle plot was positioned even closer to the *stejnegeri* parental type than the east route individual (Figure 4c). Caveats of a low sample size of three genotyped tracked birds, and potential bias because route information is limited to surviving birds, make our conclusions preliminary.

Since the initial hypothesis of hybrids taking intermediate routes between a migratory divide (Helbig, 1991), investigations in several systems have not identified particular genes as being responsible for migration direction (Delmore & Irwin, 2014; Delmore et al. 2016; Delmore et al., 2020b; Sokolovskis et al., 2023; Zając et al., 2024; Zhao et al., 2020). The genes responsible for putative trait differences might be few and camouflaged in the overall population genomic structure. In-depth bioinformatic analysis with a bigger dataset of phenotypic traits may improve our understanding of links between phenotypes and genotypes, e.g., in genome-wide association studies (GWAS) (Bossu et al., 2022). To understand the genetic basis of migration direction, future studies in the Asian stonechat system will require the collection of more migration tracking and genomic resequencing data. A Genoscape method, i.e., mapping genotypes over the geographical distribution of the breeding range and genotyping origin-unknown individuals from non-breeding sites, may be one efficient way to accumulate more data to support a GWAS analysis (Ruegg et al., 2017). Furthermore, a high-resolution annotated reference genome of stonechats should also be aimed for, in order to facilitate the annotations of candidate genes (Weissensteiner et al., 2025).

The Asian stonechat system provides further opportunities to study the relationship between migration patterns and biogeography. Irwin et al. (2005a, 2005b) demonstrated the hypothetical formation of the biogeographic pattern of the Greenish Warbler and Two-barred Warbler (*Phylloscopus plumbeitarsus*) from both a ringed speciation and a migratory divide perspective. Though not directly inferred, the formation of the migratory divide between the Greenish and Two-Barred Warblers could be assumed as a result of their ringed speciation. In the Willow Warbler system, two contact zones between the subspecies *P. t. trochilus* and *acredula* were assumed to be associated with respective migratory divides (Bensch et al., 2009). In both studies, the two closely related taxa with different migration phenotypes that came into secondary contact were assumed to experience post-zygotic reproductive isolation induced by the suboptimal intermediate route.

However, the Asian stonechat system seems not to perfectly follow the patterns described for these warbler systems. First, different to the Greenish Warbler, whose QTP population was more closely related to the western Siberian breeding population, the *przewalskii* stonechat seemed to share a closer relationship with *stejnegeri*, the eastern Siberian breeding population (Table S3). This suggests different expansion histories of breeding ranges between two taxa. An inclusion of the subspecies *indicus* (Figure 1) in the genetic study is needed to re-construct the historical expansion of stonechats in Asia. Second, the seemingly viable intermediate route phenotype, extensive hybridization, and low *F*_ST_ between *maurus* and *stejnegeri* also indicate a complex scenario, which may not be perfectly explained by a ringed speciation scenario, that *maurus* and *stejnegeri* have secondary contact where the migratory divide lies.

An alternative scenario may suggest that the migratory divide was formed *in situ*, i.e., a new migration phenotype emerged abruptly during the historical population expansion. In the best-supported *K* = 2 admixture model, the Ilchir to Heilongjiang populations were all inferred to be hybrids between *maurus* and *przewalskii* (Figures 1 and 4a); the high correlation between PC1 and PC2 with breeding longitudes between *maurus* and *stejnegeri* (Figure 5b and c) can also be explained as a single continuous population. Even though we cannot yet differentiate which scenario applies to the stonechat migratory divide, we propose that an *in situ* occurrence of a migratory divide is plausible. In fact, new migration directions can emerge in bird populations in a very short range of time, e.g., in the Richard Pipits (*Anthus richardi*), Yellow-browed Warblers (*Phylloscopus inornatus*), and Eurasian Blackcaps (Dufour et al., 2021, 2022; Helbig et al., 1994). Future studies of demographic history in Asian stonechats, including more sampling sites, e.g., from the *indicus* range, will help us to further untangle the relationship between migration phenotypes and biogeographical patterns. Studies on other species with a presumed Siberian migratory divide, where data are largely lacking, are also important to help us understand the evolution of landbird migration in Asia.

Lastly, the speciation between *maurus* and *stejnegeri* may not be only due to, or result from, the route differentiations, for instance, if the previously reported song differences between *maurus* and *stejnegeri* would indeed facilitate assortative mating (Mortega et al., 2014; Opaev et al., 2018). The plumage trait differences between *maurus* and *stejnegeri* also suggested that the gene flow between the two taxa is not unlimited. Future collaborations between taxonomists, and specialists on genomes and on migration, will help us improve our knowledge on how speciation of stonechat taxa is associated with their diversified migration behaviors.

## Conclusions

Our study revealed a diversified migration phenotype, and its associated genotypic variations, among stonechats in Asia. The differential route choices and geographical barriers may have resulted in genomic differentiation, but hybridization and survival of intermediate migration phenotypes may have dampened a speciation process and may provide sources for future evolution.

Our study complements investigations on the genetic basis of migration in a novel system, in parallel to established systems from Europe, North America, and Asia. The different expansion histories and the interaction process between barrier landscapes and evolution in different flyways could provide multiple systems to assess whether or not the evolution of migration phenotypes converged. Since the evolutionary history of migration may vary among systems, it is important to involve diverse systems, including the so far under-represented Central Asian flyways of migratory songbirds. We expect that an extended dataset on Asian stonechats could thus contribute substantially toward understanding the evolution of landbird migration.

## Supplementary Material

qrag019_Supplemental_Files

## Data Availability

The geolocator data, both the raw data and processed geolocation data, are archived in a Movebank repository: https://doi.org/10.5441/001/1.705. All the genomic re-sequencing data will be archived in the bioproject under the GenBank accession number PRJEB111081, and can be requested on demand. Codes are available at: https://github.com/ksakuras/Asian-stonechat-geolocation-and-geonomics/releases/tag/v1.0

## References

[bib1] Alexander D. H., Novembre J., Lange K. (2009). Fast model-based estimation of ancestry in unrelated individuals. Genome Research, 19(9), 1655–1664. 10.1101/gr.094052.109 19648217 PMC2752134

[bib2] Bascón-Cardozo K., Bours A., Manthey G., Durieux G., Dutheil J. Y., Pruisscher P., Liedvogel M. (2024). Fine-scale map reveals highly variable recombination rates associated with genomic features in the Eurasian blackcap. Genome Biology and Evolution, 16(1), evad233. 10.1093/gbe/evad233 38198800 PMC10781513

[bib3] Bearhop S., Fiedler W., Furness R. W., Votier S. C., Waldron S., Newton J., Bowen G. J., Berthold P., Farnsworth K. (2005). Evolution: Assortative mating as a mechanism for rapid evolution of a migratory divide. Science, 310(5747), 502–504. 10.1126/science.1115661 16239479

[bib4] Bensch S., Andersson T., Akesson S. (1999). Morphological and molecular variation across a migratory divide in willow warblers, *Phylloscopus trochilus*. Evolution, 53(6), 1925. 10.2307/2640451 28565443

[bib5] Bensch S., Caballero-López V., Cornwallis C. K., Sokolovskis K. (2023). The evolutionary history of “suboptimal” migration routes. iScience, 26(11), 108266. 10.1016/j.isci.2023.108266 38026158 PMC10663737

[bib6] Bensch S., Grahn M., Müller N., Gay L., Åkesson S. (2009). Genetic, morphological, and feather isotope variation of migratory willow warblers show gradual divergence in a ring. Molecular Ecology, 18(14), 3087–3096. 10.1111/j.1365-294X.2009.04210.x 19457197

[bib7] Bishop C. M., Spivey R. J., Hawkes L. A., Batbayar N., Chua B., Frappell P. B., Milsom W. K., Natsagdorj T., Newman S. H., Scott G. R., Takekawa J. Y., Wikelski M., Butler P. J. (2015). The roller coaster flight strategy of bar-headed geese conserves energy during Himalayan migrations. Science, 347(6219), 250–254. 10.1126/science.1258732 25593180

[bib8] Bossu C. M., Heath J. A., Kaltenecker G. S., Helm B., Ruegg K. C. (2022). Clock-linked genes underlie seasonal migratory timing in a diurnal raptor. Proceedings of the Royal Society B: Biological Sciences, 289(1974): 20212507. 10.1098/rspb.2021.2507 PMC9069262 35506230

[bib9] Caballero-López V., Bensch S. (2024). The regulatory basis of migratory behaviour in birds: Different paths to similar outcomes. Journal of Avian Biology, 2024(11–12), e03238. 10.1111/jav.03238

[bib10] Caballero-López V., Lundberg M., Sokolovskis K., Bensch S. (2021). Transposable elements mark a repeat-rich region associated with migratory phenotypes of willow warblers (*Phylloscopus trochilus*). Molecular Ecology, 31(4), 1128–1141. 10.1111/mec.16292 34837428

[bib11] Chen S. (2023). Ultrafast one-pass FASTQ data preprocessing, quality control, and deduplication using fastp. Imeta, 2(2), e107. 10.1002/imt2.107 38868435 PMC10989850

[bib12] Chen S., Zhou Y., Chen Y., Gu J. (2018). fastp: An ultra-fast all-in-one FASTQ preprocessor. Bioinformatics, 34(17), i884–i890. 10.1093/bioinformatics/bty560 30423086 PMC6129281

[bib13] Collar N. (2020). White-throated bushchat (Saxicola insignis), version 1.0. In del Hoyo J., Elliott A., Sargatal J., Christie D. A., de Juana E. (Eds.), In birds of the world. Cornell Lab of Ornithology.

[bib14] Collar N. (2021). Siberian Stonechat (*Saxicola maurus*), version 1.1. In Birds of the world. Cornell Lab of Ornithology. 10.2173/bow.sibsto1.01.1

[bib15] Danecek P., Bonfield J. K., Liddle J., Marshall J., Ohan V., Pollard M. O., Li H. (2021). Twelve years of SAMtools and BCFtools. GigaScience, 10(2), giab008. 10.1093/gigascience/giab008 33590861 PMC7931819

[bib16] Delmore K., Illera J. C., Pérez-Tris J., Segelbacher G., Lugo Ramos J. S., Durieux G., Ishigohoka J., Liedvogel M. (2020a). The evolutionary history and genomics of European blackcap migration. eLife, 9, 1–24. 10.7554/eLife.54462 PMC7173969 32312383

[bib17] Delmore K. E., Irwin D. E. (2014). Hybrid songbirds employ intermediate routes in a migratory divide. Ecology Letters, 17(10), 1211–1218. 10.1111/ele.12326 25040456

[bib18] Delmore K. E., Toews D. P. L., Germain R. R., Owens G. L., Irwin D. E. (2016). The genetics of seasonal migration and plumage color. Current Biology, 26(16), 2167–2173. 10.1016/j.cub.2016.06.015 27476599

[bib19] Delmore K. E., van Doren B. M., Conway G. J., Curk T., Garrido-Garduño T., Germain R. R., Hasselmann T., Hiemer D., van der Jeugd H. P., Justen H., Lugo Ramos J. S., Maggini I., Meyer B. S., Phillips R. J., Remisiewicz M., Roberts G. C. M., Sheldon B. C., Vogl W., Liedvogel M. (2020b). Individual variability and versatility in an eco-evolutionary model of avian migration. Proceedings of the Royal Society B: Biological Sciences, 287(1938), 20201339. 10.1098/rspb.2020.1339 PMC7735267 33143577

[bib20] Delmore K. E., Van Doren B. M., Ullrich K., Curk T., van der Jeugd H. P., Liedvogel M. (2023). Structural genomic variation and migratory behavior in a wild songbird. Evolution Letters, 7(6), 401–412. 10.1093/evlett/qrad040 38045725 PMC10693001

[bib21] Dong S., Shang Z., Gao J., Boone R. B. (2020). Enhancing sustainability of grassland ecosystems through ecological restoration and grazing management in an era of climate change on Qinghai-Tibetan Plateau. Agriculture, Ecosystems & Environment, 287, 106684. 10.1016/j.agee.2019.106684

[bib22] Drovetski S. V., Zink R. M., Rohwer S., Fadeev I. V., Nesterov E. V., Karagodin I., Koblik E. A., Red’kin Y. A. (2004). Complex biogeographic history of a Holarctic passerine. Proceedings of the Royal Society B: Biological Sciences, 271(1538), 545–551. 10.1098/rspb.2003.2638 PMC1691619 15129966

[bib23] Dufour P., Åkesson S., Hellström M., Hewson C., Lagerveld S., Mitchell L., Chernetsov N., Schmaljohann H., Crochet P. A. (2022). The yellow-browed warbler (*Phylloscopus inornatus*) as a model to understand vagrancy and its potential for the evolution of new migration routes. Movement Ecology, 10(1), 1–15. 10.1186/s40462-022-00345-2 36517925 PMC9753335

[bib24] Dufour P., de Franceschi C., Doniol-Valcroze P., Jiguet F., Guéguen M., Renaud J., Crochet P. A. (2021). A new westward migration route in an Asian passerine bird. Current Biology, 31(24), 5590–5596. 10.1016/j.cub.2021.09.086 34687610

[bib25] Galtbalt B., Batbayar N., Sukhbaatar T., Vorneweg B., Heine G., Müller U., Wikelski M., Klaassen M. (2022). Differences in on-ground and aloft conditions explain seasonally different migration paths in Demoiselle crane. Movement Ecology, 10(1), 1–11. 10.1186/s40462-022-00302-z 35101131 PMC8805327

[bib26] Gwinner E., Dittami J. (1990). Endogenous reproductive rhythms in a tropical bird. Science, 249(4971), 906–908. 10.1126/science.249.4971.906 17773105

[bib27] Hawkes L. A., Balachandran S., Batbayar N., Butler P. J., Frappell P. B., Milsom W. K., Tseveenmyadag N., Newman S. H., Scott G. R., Sathiyaselvam P., Takekawa J. Y., Wikelski M., Bishop C. M. (2011). The trans-Himalayan flights of bar-headed geese (*Anser indicus*). Proceedings of the National Academy of Sciences of the United States of America, 108(23), 9516–9519. 10.1073/pnas.1017295108 21628594 PMC3111297

[bib28] Heim W., Heim R. J., Beermann I., Burkovskiy O. A., Gerasimov Y., Ktitorov P., Kamp J. (2020). Using geolocator tracking data and ringing archives to validate citizen-science based seasonal predictions of bird distribution in a data-poor region. Global Ecology and Conservation, 24, e01215. 10.1016/j.gecco.2020.e01215

[bib29] Heim W., Pedersen L., Heim R., Kamp J., Smirenski S. M., Thomas A., Tøttrup A. P., Thorup K. (2018). Full annual cycle tracking of a small songbird, the Siberian rubythroat *Calliope calliope*, along the East Asian flyway. Journal of Ornithology, 159(4), 893–899. 10.1007/s10336-018-1562-z

[bib30] Helbig A. (1996). Genetic basis, mode of inheritance and evolutionary changes of migratory directions in palearctic warblers (Aves: Sylviidae). Journal of Experimental Biology, 199(1), 49–55. 10.1242/jeb.199.1.49 9317319

[bib31] Helbig A. J. (1991). Inheritance of migratory direction in a bird species: A cross-breeding experiment with SE- and SW-migrating blackcaps (*Sylvia atricapilla*). Behavioral Ecology and Sociobiology, 28(1), 9–12. 10.1007/BF00172133

[bib32] Helbig A. J., Berthold P., Mohr G., Querner U. (1994). Inheritance of a novel migratory direction in central european blackcaps. Die Naturwissenschaften, 81(4), 184–186. 10.1007/s001140050055

[bib33] Hellström M., Norevik G. (2014). The uppertail-covert pattern of “Stejneger’s stonechat.”. British Birds, 107(11), 692–700.

[bib34] Hellström M., Waern M. (2023). Identification of extralimital Siberian and Amur stonechats. British Birds, 116(1), 41–53.

[bib35] Helm B., Gwinner E. (2006). Migratory restlessness in an equatorial nonmigratory bird. PLoS Biology, 4(4), e110. 10.1371/journal.pbio.0040110 16555925 PMC1420642

[bib36] Helm B., Schwabl I., Gwinner E. (2009). Circannual basis of geographically distinct bird schedules. Journal of Experimental Biology, 212(9), 1259–1269. 10.1242/jeb.025411 19376946

[bib37] Illera J. C., Richardson D. S., Helm B., Atienza J. C., Emerson B. C. (2008). Phylogenetic relationships, biogeography and speciation in the avian genus *Saxicola*. Molecular Phylogenetics and Evolution, 48(3), 1145–1154. 10.1016/j.ympev.2008.05.016 18571939

[bib38] Irwin D. E., Bensch S., Irwin J. H., Price T. D. (2005a). Speciation by distance in a ring species. Science, 307(5708), 414–416. 10.1126/science.1105201 15662011

[bib39] Irwin D. E., Irwin J. H., Greenberg R., Marra P. P. (2005b). Siberian migratory divides: The role of seasonal migration in speciation. In R. Greenberg & P. Marra (Eds.), Birds of two worlds: The ecology and evolution of migration (pp. 27–40). Johns Hopkins University Press.

[bib40] Justen H., Delmore K. E. (2022). The genetics of bird migration. Current Biology, 32(20), R1144–R1149. 10.1016/j.cub.2022.07.008 36283382

[bib41] Justen H., Hasselmann T., Illera J. C., Delmore K. E., Serrano D., Flinks H., Senzaki M., Kawamura K., Helm B., Liedvogel M. (2022). Population-specific association of Clock gene polymorphism with annual cycle timing in stonechats. Scientific Reports, 12(1), 7947. 10.1038/s41598-022-11158-z 35562382 PMC9106710

[bib42] Justen H. C., Easton W. E., Delmore K. E. (2024). Mapping seasonal migration in a songbird hybrid zone—heritability, genetic correlations, and genomic patterns linked to speciation. Proceedings of the National Academy of Sciences of the United States of America, 121(18), e2313442121. 10.1073/pnas.2313442121 38648483 PMC11067064

[bib43] Kumar N., Gupta U., Jhala Y. V., Qureshi Q., Gosler A. G., Sergio F. (2020). GPS-telemetry unveils the regular high-elevation crossing of the Himalayas by a migratory raptor: Implications for definition of a “Central Asian Flyway.” Scientific Reports, 10(1), 1–9. 10.1038/s41598-020-72970-z 32994476 PMC7524735

[bib44] Lawson D. J., Van Dorp L., Falush D. (2018). A tutorial on how not to over-interpret STRUCTURE and ADMIXTURE bar plots. Nature Communications, 9(1), 3258. 10.1038/s41467-018-05257-7 PMC6092366 30108219

[bib45] Liedvogel M., Åkesson S., Bensch S. (2011). The genetics of migration on the move. Trends in Ecology & Evolution, 26(11), 561–569. 10.1016/j.tree.2011.07.009 21862171

[bib46] Lindström Å., Alerstam T., Andersson A., Bäckman J., Bahlenberg P., Bom R., Ekblom R., Klaassen R. H. G., Korniluk M., Sjöberg S., Weber J. K. M. (2021). Extreme altitude changes between night and day during marathon flights of great snipes. Current Biology, 31(15), 3433–3439. 10.1016/j.cub.2021.05.047 34197730

[bib47] Lisovski S., Bauer S., Briedis M., Davidson S. C., Dhanjal-Adams K. L., Hallworth M. T., Karagicheva J., Meier C. M., Merkel B., Ouwehand J., Pedersen L., Rakhimberdiev E., Roberto-Charron A., Seavy N. E., Sumner M. D., Taylor C. M., Wotherspoon S. J., Bridge E. S. (2020). Light-level geolocator analyses: A user’s guide. Journal of Animal Ecology, 89(1), 221–236. 10.1111/1365-2656.13036 31190329

[bib48] Liu S., Cheng F., Dong S., Zhao H., Hou X., Wu X. (2017). Spatiotemporal dynamics of grassland aboveground biomass on the Qinghai-Tibet Plateau based on validated MODIS NDVI. Scientific Reports, 7(1), 4182. 10.1038/s41598-017-04038-4 28646198 PMC5482894

[bib49] Lundberg M., Liedvogel M., Larson K., Sigeman H., Grahn M., Wright A., Åkesson S., Bensch S. (2017). Genetic differences between willow warbler migratory phenotypes are few and cluster in large haplotype blocks. Evolution Letters, 1(3), 155–168. 10.1002/evl3.15 30283646 PMC6121796

[bib50] McKinnon E. A., Love O. P. (2018). Ten years tracking the migrations of small landbirds: Lessons learned in the golden age of bio-logging. The Auk, 135(4), 834–856. 10.1642/auk-17-202.1

[bib51] Mi C., Li X., Huettmann F., Goroshko O., Guo Y. (2022). Time and energy minimization strategy codetermine the loop migration of demoiselle cranes around the Himalayas. Integrative Zoology, 17(5), 715–730. 10.1111/1749-4877.12632 35060680

[bib52] Mölder F., Jablonski K. P., Letcher B., Hall M. B., Tomkins-Tinch C. H., Sochat V., Köster J. (2021). Sustainable data analysis with Snakemake. F1000Research, 10, 33. 34035898 10.12688/f1000research.29032.1 PMC8114187

[bib53] Mortega K. G., Flinks H., Helm B. (2014). Behavioural response of a migratory songbird to geographic variation in song and morphology. Frontiers in Zoology, 11(1), 85. 10.1186/s12983-014-0085-6 25484906 PMC4256809

[bib54] Opaev A., Red’kin Y., Kalinin E., Golovina M. (2018). Species limits in Northern Eurasian taxa of the common stonechats, *Saxicola torquatus* complex (Aves: Passeriformes, Muscicapidae). Vertebrate Zoology, 68(3), 199–211. 10.3897/vz.68.e31606

[bib55] Prins H. H. T., Namgail T. (2017). Bird migration across the himalayas: Wetland functioning amidst mountains and glaciers. Cambridge University Press. 10.1017/9781316335420

[bib56] Proud D. (1949). Some notes on the birds of the Nepal Valley. Journal of the Bombay Natural History Society, 48(4), 696–719.

[bib57] Purcell S., Neale B., Todd-Brown K., Thomas L., Ferreira M. A., Bender D., Sham P. C. (2007). PLINK: A tool set for whole-genome association and population-based linkage analyses. The American Journal of Human Genetics, 81(3), 559–575. 10.1086/519795 17701901 PMC1950838

[bib58] R Core Team . (2024). R: A Language and Environment for Statistical Computing. R Foundation for Statistical Computing, Vienna, Austriahttps://www.R-project.org/

[bib59] Ruegg K., Anderson E. C., Boone J., Pouls J., Smith T. B. (2014). A role for migration-linked genes and genomic islands in divergence of a songbird. Molecular Ecology, 23(19), 4757–4769. 10.1111/mec.12842 24954641

[bib60] Ruegg K. C., Anderson E. C., Harrigan R. J., Paxton K. L., Kelly J. F., Moore F., Smith T. B. (2017). Genetic assignment with isotopes and habitat suitability (GAIAH), a migratory bird case study. Methods in Ecology and Evolution, 8(10), 1241–1252. 10.1111/2041-210X.12800

[bib61] Ruegg K. C., Smith T. B. (2002). Not as the crow flies: A historical explanation for circuitous migration in Swainson’s thrush (*Catharus ustulatus*). Proceedings of the Royal Society of London. Series B: Biological Sciences, 269(1498), 1375–1381. 10.1098/rspb.2002.2032 PMC1691041 12079661

[bib62] Scordato E. S. C., Smith C. C. R., Semenov G. A., Liu Y., Wilkins M. R., Liang W., Rubtsov A., Sundev G., Koyama K., Turbek S. P., Wunder M. B., Stricker C. A., Safran R. J. (2020). Migratory divides coincide with reproductive barriers across replicated avian hybrid zones above the Tibetan Plateau. Ecology Letters, 23(2), 231–241. 10.1111/ele.13420 31746098

[bib63] Sjöberg S., Malmiga G., Nord A., Andersson A., Bäckman J., Tarka M., Willemoes M., Thorup K., Hansson B., Alerstam T., Hasselquist D. (2021). Extreme altitudes during diurnal flights in a nocturnal songbird migrant. Science, 372(6542), 646–648. 10.1126/science.abe7291 33958477

[bib64] Sokolovskis K., Lundberg M., Åkesson S., Willemoes M., Zhao T., Caballero-López V., Bensch S. (2023). Migration direction in a songbird explained by two loci. Nature Communications, 14(1), 1–6. 10.1038/s41467-023-35788-7 PMC9834303 36631459

[bib65] Spierenburg P. (2005). Birds in bhutan: Status and distribution. Oriental Bird Club.

[bib66] Strubbe S., Bourski O., Amshoff J., Meinken M., Tarasov V., Volkova V., Heim W. (2025). Tracking the migration of a Bluethroat *Luscinia svecica svecica* from central Siberia. Journal of Ornithology, 166, 869–874. 10.1007/s10336-025-02270-8

[bib67] Taylor R. S., Friesen V. L. (2017). The role of allochrony in speciation. Molecular Ecology, 26(13), 3330–3342. 10.1111/mec.14126 28370658

[bib68] Turbek S. P., Schield D. R., Scordato E. S. C., Contina A., Da X. W., Liu Y., Liu Y., Pagani-Núñez E., Ren Q. M., Smith C. C. R., Stricker C. A., Wunder M., Zonana D. M., Safran R. J. (2022). A migratory divide spanning two continents is associated with genomic and ecological divergence. Evolution, 76(4), 722–736. 10.1111/evo.14448 35166383

[bib69] Tyler S., de Juana E. (2020). Rosy Pipit (*Anthus roseatus*), version 1.0. In del Hoyo J., Elliott A., Sargatal J., Christie D. A., de Juana E. (Eds.), Birds of the world. Cornell Lab of Ornithology.

[bib70] Tyler S., de Juana E., Kirwan G. M. (2020). Blyth’s pipit (Anthus godlewskii), version 1.0. In del Hoyo J., Elliott A., Sargatal J., Christie D. A., de Juana E. (Eds.), Birds of the world. Cornell Lab of Ornithology.

[bib71] Urquhart E. (2010). Stonechats. A&C Black.

[bib72] van Bemmelen R. S. A., Kolbeinsson Y., Ramos R., Gilg O., Alves J. A., Smith M., Schekkerman H., Lehikoinen A., Petersen I. K., Pórisson B., Sokolov A. A., Välimäki K., van der Meer T., David Okill J., Bolton M., Moe B., Hanssen S. A., Bollache L., Petersen A., … Tulp I. (2019). A migratory divide among red-necked phalaropes in the Western Palearctic reveals contrasting migration and wintering movement strategies. Frontiers in Ecology and Evolution, 7, 86. 10.3389/fevo.2019.00086

[bib73] van Doren B. M., Campagna L., Helm B., Illera J. C., Lovette I. J., Liedvogel M. (2017a). Correlated patterns of genetic diversity and differentiation across an avian family. Molecular Ecology, 26(15), 3982–3997. 10.1111/mec.14083 28256062

[bib74] van Doren B. M., Liedvogel M., Helm B. (2017b). Programmed and flexible: Long-term Zugunruhe data highlight the many axes of variation in avian migratory behaviour. Journal of Avian Biology, 48(1), 155–172. 10.1111/jav.01348

[bib75] Vasimuddin M., Misra S., Li H., Aluru S. (2019). Efficient architecture-aware acceleration of BWA-MEM for multicore systems. In Proceedings of the 2019 IEEE international parallel and distributed processing symposium (IPDPS) (pp. 314–324.). IEEE. 10.1109/IPDPS.2019.00041

[bib76] Veen T., Borge T., Griffith S. C., Saetre G. P., Bures S., Gustafsson L., Sheldon B. C. (2001). Hybridization and adaptive mate choice in flycatchers. Nature, 411(6833), 45–50. 10.1038/35075000 11333971

[bib77] Veen T., Svedin N., Forsman J. T., Hjernquist M. B., Qvarnström A., Hjernquist K. A. T., Klaassen M. (2007). Does migration of hybrids contribute to post-zygotic isolation in flycatchers?. Proceedings of the Royal Society B: Biological Sciences, 274(1610), 707–712. 10.1098/rspb.2006.0058 PMC2197218 17254995

[bib78] Weissensteiner M. H., Delmore K., Peona V., Lugo Ramos J. S., Arnaud G., Blas J., Liedvogel M. (2025). Combining individual-based radio-tracking with whole-genome sequencing data reveals candidate for genetic basis of partial migration in a songbird. Ecology and Evolution, 15(1), e70800. 10.1002/ece3.70800 39803194 PMC11717897

[bib79] Wiens B (2025). triangulaR: Calculate hybrid index and heterozygosity from SNP data. R package version 0.0.1. https://github.com/omys-omics/triangulaR

[bib80] Wiens B. J., DeCicco L. H., Colella J. P. (2025). triangulaR: An R package for identifying AIMs and building triangle plots using SNP data from hybrid zones. Heredity, 134, 251–262. 40216927 10.1038/s41437-025-00760-2 PMC12056084

[bib81] Yamaura Y., Schmaljohann H., Lisovski S., Senzaki M., Kawamura K., Fujimaki Y., Nakamura F. (2017). Tracking the Stejneger’s stonechat *Saxicola stejnegeri* along the East Asian-Australian Flyway from Japan via China to southeast Asia. Journal of Avian Biology, 48(2), 197–202. 10.1111/jav.01054

[bib82] Yamaura Y., Schmaljohann H., Unno A., Kawamura K., Kitazawa M., Sato S., Senzaki M. (2026). Loop vs. same route migrations of two songbird species between Japan and Southeast Asia. Journal of Ornithology, 167, 71–83.

[bib83] Yu X., Song G., Wang H., Wei Q., Jia C., Lei F. (2024). Migratory flyways and connectivity of Brown Headed Gulls (*Chroicocephalus brunnicephalus*) revealed by GPS tracking. Global Ecology and Conservation, 56, e03340. 10.1016/j.gecco.2024.e03340

[bib84] Zając J. A., Neubauer G., Korner-Nievergelt F., Zagalska-Neubauer M. (2024). Unravelling intermediate migration patterns in gull hybrids: Insights from ring re-encounters. Scientific Reports, 14(1), 27050. 10.1038/s41598-024-77476-6 39511258 PMC11543659

[bib85] Zhao T., Heim W., Nussbaumer R., van Toor M., Zhang G., Andersson A., Bäckman J., Liu Z., Song G., Hellström M., Roved J., Liu Y., Bensch S., Wertheim B., Lei F., Helm B. (2024). Seasonal migration patterns of Siberian Rubythroat (*Calliope calliope*) facing the Qinghai-Tibet Plateau. Movement Ecology, 12(1), 54. 10.1186/s40462-024-00495-5 39090724 PMC11295652

[bib86] Zhao T., Ilieva M., Larson K., Lundberg M., Neto J. M., Sokolovskis K., Åkesson S., Bensch S. (2020). Autumn migration direction of juvenile willow warblers (*Phylloscopus t. trochilus* and *P. t. acredula*) and their hybrids assessed by qPCR SNP genotyping. Movement Ecology, 8(1), 1–10. 10.1186/s40462-020-00209-7 32514357 PMC7257155

[bib87] Zink R. M., Pavlova A., Drovetski S., Wink M., Rohwer S. (2009). Taxonomic status and evolutionary history of the *Saxicola torquata* complex. Molecular Phylogenetics and Evolution, 52(3), 769–773. 10.1016/j.ympev.2009.05.016 19464380

